# Severe Encrustation, Fragmentation, and Urethral Extrusion of a Forgotten Ureteral Stent Managed With a Minimally Invasive Approach

**DOI:** 10.1002/ccr3.73572

**Published:** 2026-09-21

**Authors:** Mostafa Amro, Asem Afana, Ibraheem Absah, Hossam Salameh, Alaa Alazzeh

**Affiliations:** ^1^ Department of Medicine An‐Najah National University Nablus Palestine; ^2^ Department of Urology Al‐Ahli Hospital Hebron Palestine

**Keywords:** DJ stent, fragmentation, urethral extrusion, urinary tract infection

## Abstract

We present a rare case of a severely encrusted DJ stent that remained in situ for 3.5 years, with an unusual presentation, successfully managed with minimally invasive techniques despite differing views in the literature.

## Key Clinical Message

Severely encrusted and fragmented forgotten ureteral stents can, in carefully selected cases, be successfully managed with a single‐session minimally invasive approach; however, strict follow‐up and stent‐tracking systems are essential to prevent such potentially serious complications.

## 1 Introduction

Double‐J (DJ) ureteral stents are widely used in the world of urology, primarily to relieve ureteral obstruction and ensure urinary drainage, especially following endourological procedures [1]. While generally considered safe, indwelling DJ stents are associated with both short‐term complications, such as urinary tract infections (UTIs), hematuria, and pain, and serious long‐term risks if forgotten [1]. Prolonged indwelling increases the likelihood of encrustation, fragmentation, infection, migration, and ureteral injury.

Current clinical guidelines recommend the removal or replacement of DJ stents after 3–6 months to avoid complications, with many authorities supporting removal at or below 3 months in uncomplicated cases [2]. Retained stents beyond this period become exponentially more complex to manage due to calcification and encrustation [3].

The management of the forgotten DJ stent can be challenging, and may involve a number of interventions, including extracorporeal shock wave lithotripsy (ESWL), percutaneous nephrolithotomy (PCNL), ureteroscopy with laser lithotripsy, or even open surgery in severe cases [3]. The choice of management depends on several factors, such as the location and degree of encrustation, the condition of the stent (e.g., intact, fragmented, or migrated), and patient comorbidities.

This case report has been reported in line with the SCARE criteria [4].

## 2 Case History/Examination

A 47‐year‐old female patient presented to the urology clinic with a complaint of passing a foreign object during urination 3 days prior to presentation. She had a history of a right PCNL performed at another Facility 3.5 years ago for a renal stone, during which a DJ stent was inserted. According to the patient, she was not informed about the stent placement at the time and was subsequently lost to follow‐up. This lack of patient awareness, combined with the absence of a structured follow‐up system, likely contributed to prolonged stent retention.

**FIGURE 1:**
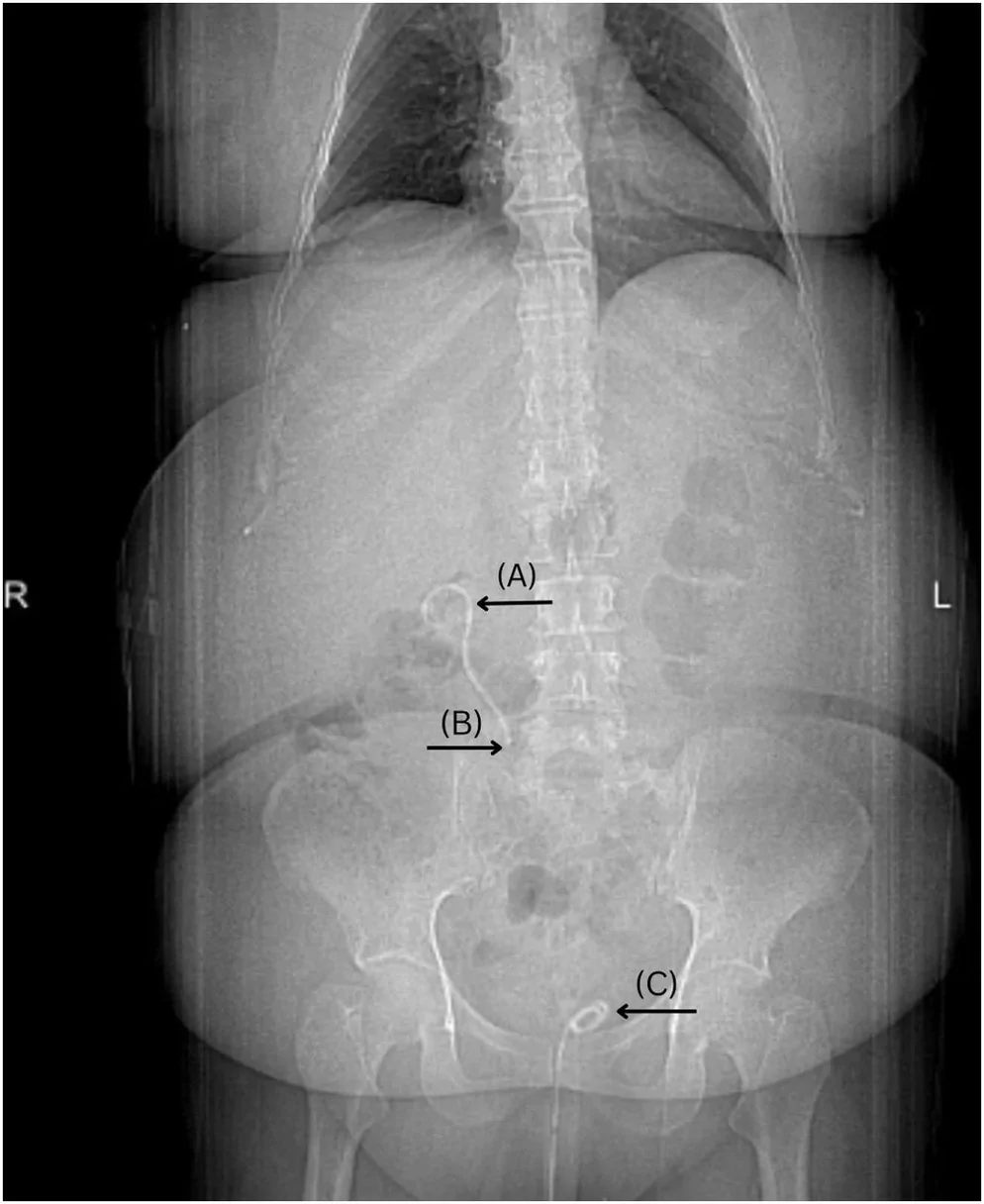
Kidney, ureter, and bladder (KUB) radiograph demonstrating a fragmented right‐sided DJ stent. (A) Proximal coil located within the right renal pelvis. (B) Discontinuity along the ureter consistent with stent fragmentation. (C) Distal coil visualized within the urinary bladder.

Over the past 3 years, the patient experienced multiple episodes of urinary tract infections (UTIs), which were treated with antibiotics without further investigations into the underlying cause. She otherwise remained in good health until 3 days before presentation, when she experienced a sudden onset of a foreign object protruding from her external genitalia. She sought medical attention at the primary health care center and was referred to our urology clinic for further evaluation.

On physical examination, the patient was afebrile with stable vital signs. Mild tenderness was noted in the right costovertebral angle. An encrusted portion of the DJ stent was visibly protruding from the urethra. Urinalysis revealed numerous pus cells and red blood cells. The patient denied any preceding lower urinary tract symptoms, fever, or recent antibiotic use prior to presentation. Routine preoperative laboratory investigations were performed, and renal function tests were within normal limits; however, detailed inflammatory markers and urine culture results were not available at the time of documentation. There was no clinically significant hydronephrosis identified; however, the limited non‐contrast CT reported mild pelvic and upper calyceal dilatation.

## 3 Differential Diagnosis, Investigations and Treatment

Differential diagnoses included retained ureteral stent, bladder calculus, and other intravesical foreign bodies.

A kidney, ureter, and bladder (KUB) X‐ray (Figure 1) demonstrated a fragmented DJ stent. The proximal segment remained in the right kidney, with its distal tip extending into the right ureter at the level of the L5 vertebra. The distal segment was located in the urinary bladder, with its proximal end protruding through the urethra. A limited non‐contrast CT scan was also performed and confirmed the broken right DJ stent, with the proximal segment remaining within the right kidney and extending to the level of L5, while the distal segment was located in the urinary bladder and extended through the urethra. Mild pelvic and upper calyceal dilatation was also reported.

**FIGURE 2:**
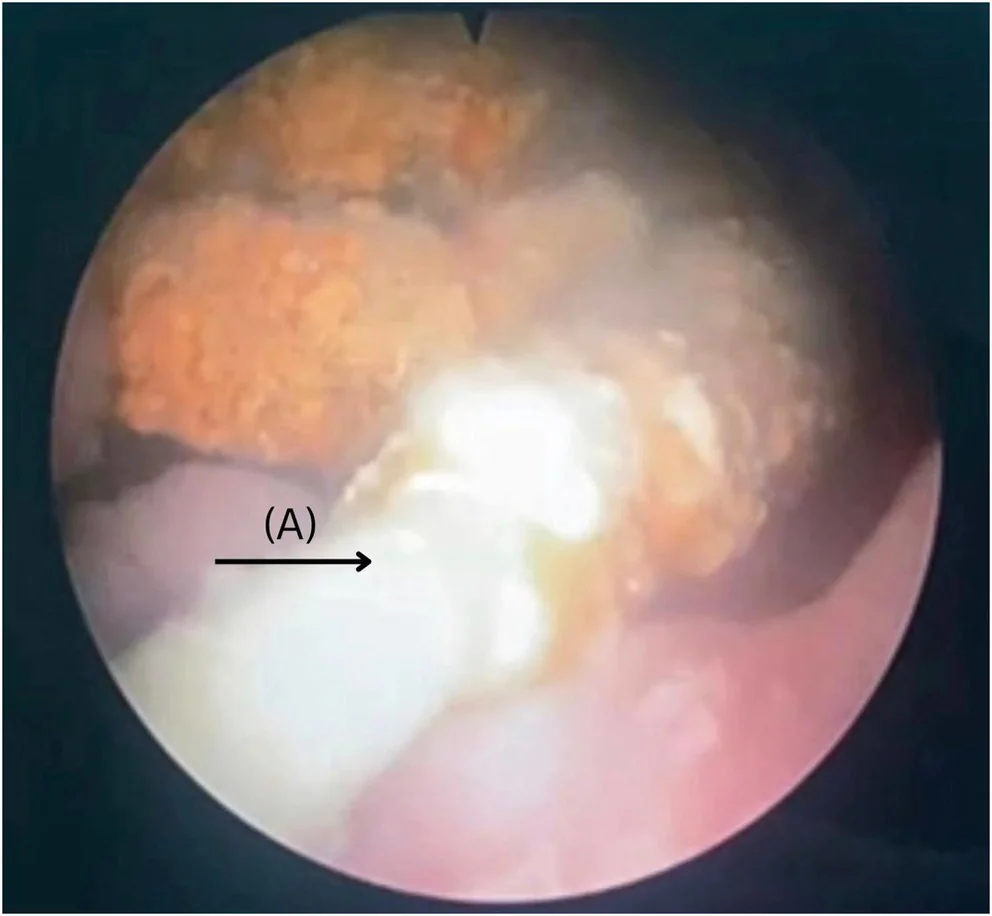
Cystoscopic view demonstrating the encrusted distal segment of the fragmented DJ stent within the urinary bladder. (A) Encrusted stent fragment embedded within calcified deposits.

The patient was diagnosed with a missed, encrusted, and fragmented DJ stent. She was taken to the operating room, where cystoscopy under general anesthesia revealed the encrusted distal segment of the broken segment lodged in the urinary bladder (Figure 2). Ureteroscopy with laser lithotripsy was then performed to fragment and remove the encrustation around the length of the broken DJ stent, which was then removed completely. Ureteral patency was confirmed, and a new 6F DJ stent was inserted under fluoroscopic guidance.

## 4 Conclusion and Results (Outcome and Follow‐Up)

The procedure was completed without complications. The patient was observed in the surgical ward and discharged the following day in good general condition, hemodynamically stable, tolerating an oral diet, and passing stool and urine normally. She was advised to increase her fluid intake and was scheduled for a follow‐up visit the next week. She was also informed of the need to return in 4–6 weeks for the removal of the new DJ stent. At the time of manuscript preparation, follow‐up information regarding the removal of the newly inserted DJ stent and the patient's subsequent clinical course was not yet available. This limitation is acknowledged in the Discussion.

A structured timeline of the patient's clinical course is presented in Table 1.

**TABLE 1:** Timeline of clinical events.

Time	Clinical event
3.5 years prior	Right PCNL with DJ stent insertion
The following years before presentation	Recurrent UTIs treated symptomatically
3 days before presentation	Foreign body protrusion from urethra
Day of presentation	Clinical evaluation and KUB imaging
Same admission	Endoscopic removal and new DJ stent insertion
Postoperative Day 1	Discharged in stable condition
Follow‐up planned	Stent removal after 4–6 weeks (follow‐up data not available at the time of manuscript preparation)

## 5 Discussion

Forgotten ureteral stents are associated with a spectrum of complications, the severity of which correlates strongly with the duration of indwelling time. Previous studies have demonstrated that the risk of encrustation increases significantly after 6 weeks, while prolonged retention beyond 12 weeks markedly raises the likelihood of fragmentation and complex stone formation [5]. In the present case, the stent remained in situ for over 42 months, resulting in severe encrustation and spontaneous fragmentation.

Stent fragmentation is an uncommon but well‐documented complication. In rare instances, this may lead to “stenturia,” a phenomenon in which a stent fragment migrates distally and extrudes through the urethra. Such presentations are typically associated with significant encrustation or prior interventions such as ESWL [6]. However, in this case, extrusion occurred spontaneously without any preceding procedure, making it a particularly unusual presentation.

To better characterize the severity of retained stents and guide management, several grading systems have been proposed, including the FECal classification and the KUB grading system [7, 8]. Based on the radiographic appearance of encrustation involving both the proximal and distal segments, this case may retrospectively correspond to a high‐grade classification (FECal Grade IV–V); however, formal scoring was not performed prospectively, and this classification should be interpreted with caution. Similarly, applying the KUB grading system retrospectively suggests a complex case, although this grading was not used to guide real‐time management decisions.

Management strategies for severely encrusted and fragmented stents are highly variable and depend on multiple factors, including the location and extent of encrustation, stent integrity, and patient clinical status. Many cases reported in the literature, particularly those with prolonged indwelling times, have required combined approaches such as PCNL, cystolitholapaxy, and ureteroscopy with laser lithotripsy, often performed over multiple sessions [3]. In contrast, the present case was successfully managed in a single session using ureteroscopy and laser lithotripsy, allowing complete removal of the stent without the need for more invasive procedures.

The decision to pursue a single‐session minimally invasive approach was guided by several factors, including the patient's overall clinical stability at presentation, absence of acute complications, and favorable endoscopic accessibility of the fragmented stent components. This approach avoided multiple procedures in the present patient, minimized surgical morbidity, and resulted in a short hospital stay. However, this should not be generalized to all complex retained stents, many of which will still require staged or multimodal management as described in the literature [3]; rather, this case illustrates that carefully selected patients with favorable clinical and anatomical characteristics may be amenable to less invasive strategies.

This report has several limitations. A limited non‐contrast CT scan was obtained during the patient's evaluation; however, the CT was not incorporated as an imaging figure in this report. Composition analysis of the encrustation and stone material was not performed, as fragments were not sent for laboratory analysis following endoscopic fragmentation. Additionally, as this report was prepared shortly after the index procedure, outcome data regarding removal of the newly inserted stent and any recurrence of urinary tract infection during the planned 4–6 week follow‐up period were not yet available at the time of writing.

Finally, this case emphasizes the critical importance of patient education and systematic follow‐up. Failure to inform the patient about the presence of a ureteral stent was a key contributing factor to prolonged retention. Similar findings have been reported in the literature, where lack of patient awareness accounts for a substantial proportion of forgotten stents [9]. The implementation of stent‐tracking systems and improved discharge counseling may play a crucial role in preventing such avoidable complications.

## Author Contributions

**Mostafa Amro:** conceptualization, investigation, visualization, writing – original draft. **Asem Afana:** writing – original draft. **Ibraheem Absah:** writing – original draft. **Hossam Salameh:** visualization, writing – original draft. **Alaa Alazzeh:** investigation, resources, supervision, writing – review and editing.

## Funding

The authors have nothing to report.

## Consent

Written informed consent from the patient was obtained for publication of this case report and any accompanying images. A copy of the written consent is available for review by the Editor‐in‐Chief of this journal on request.

## Conflicts of Interest

The authors declare no conflicts of interest.

## Data Availability

The data that support the findings of this study are available from the corresponding author upon reasonable request.
